# Sample size calculations in pediatric clinical trials conducted in an ICU: a systematic review

**DOI:** 10.1186/1745-6215-15-274

**Published:** 2014-07-08

**Authors:** Stavros Nikolakopoulos, Kit C B Roes, Johanna H van der Lee, Ingeborg van der Tweel

**Affiliations:** 1 Department of Biostatistics, Julius Center for Health Sciences and Primary Care, University Medical Center Utrecht, Str. 6.131, PO Box 85500, 3508 Utrecht, GA, The Netherlands; 2 Clinical Research Unit, Woman-Child Center, Academic Medical Center, University of Amsterdam, Amsterdam, The Netherlands

**Keywords:** clinical trials, sample size, power, standard deviation, event rate, study design

## Abstract

At the design stage of a clinical trial, several assumptions have to be made. These usually include guesses about parameters that are not of direct interest but must be accounted for in the analysis of the treatment effect and also in the sample size calculation (nuisance parameters, e.g. the standard deviation or the control group event rate). We conducted a systematic review to investigate the impact of misspecification of nuisance parameters in pediatric randomized controlled trials conducted in intensive care units. We searched MEDLINE through PubMed. We included all publications concerning two-arm RCTs where efficacy assessment was the main objective. We included trials with pharmacological interventions. Only trials with a dichotomous or a continuous outcome were included. This led to the inclusion of 70 articles describing 71 trials. In 49 trial reports a sample size calculation was reported. Relative misspecification could be calculated for 28 trials, 22 with a dichotomous and 6 with a continuous primary outcome. The median [inter-quartile range (IQR)] overestimation was 6.9 [-12.1, 57.8]% for the control group event rate in trials with dichotomous outcomes and -1.5 [-15.3, 5.1]% for the standard deviation in trials with continuous outcomes. Our results show that there is room for improvement in the clear reporting of sample size calculations in pediatric clinical trials conducted in ICUs. Researchers should be aware of the importance of nuisance parameters in study design and in the interpretation of the results.

## Review

### Introduction

In randomized controlled trials (RCTs), a priori sample size calculations aim at enrolling sufficient participants to detect a clinically relevant treatment effect. Including too many participants may expose some to an inferior treatment unnecessarily. Including too few may make the likelihood of reaching a definite conclusion too small. The importance of adequate sample size calculations has been widely stressed in the biomedical literature [1-4], including internationally recognized guidelines [5-7]. Most sample size calculations are easily conducted nowadays using specialized software.

In recent years, increasing attention has been given to pediatric RCTs [8-13] for pharmacological interventions due to the fact that many drugs used in children have not (yet) been tested [14]. Drug regulatory agencies implemented guidance for sponsors to promote drug research in children, leading to more trials being designed and conducted [15]. Recruitment difficulties [16,17] and ethical considerations [18,19] make pediatric trials more challenging, especially with critically ill children, e.g. children being treated in ICUs. In such cases, the importance of a rigorously designed RCT is stressed.

In the design phase of an RCT, the sample size is calculated based on the primary outcome variable. Sample size depends on parameters that are estimated or assumed, in addition to the set criteria of type I error and power. In addition to the clinically relevant treatment effect to be detected, assumptions need to be made about so-called *nuisance parameters* (NPs). A NP is a parameter that is not of direct interest but must be accounted for in the analysis of the treatment effect and thus also in the sample size calculation. Examples of NPs are the event rate in the control group (control group event rate, CER) when the clinical outcome of interest is dichotomous and the standard deviation (SD, assumed equal across groups), when the clinical outcome is continuous.

The value of a NP substantially affects the sample size calculation; therefore the value assumed should be as reliable as possible. Of course, the observed value once the trial is completed will differ from the assumed value at the design stage. If the assumed value is different from the (unknown) population value, we refer to it as the *misspecification* of the nuisance parameter. Misspecification can have serious consequences for the actual power of the trial and the smallest possible effect size that can be detected.

When a sample size calculation is performed, the value of the NP used corresponds to its assumed *population* value. Therefore, misspecification can be shown on a per trial basis in terms of statistical significance. That is, test whether the observed value is significantly different from the assumed population value. However, our focus will be on *systematic misspecification*. We are interested in exploring whether there is systematic over- or underestimation of NPs in a specific population of pediatric RCTs, and what the consequences of such a systematic misspecification are on the design aspects of these RCTs and the inference that can be drawn from them. There are various ways to arrive at an assumption about the value of a NP. One can estimate it based on data from earlier trials or other types of studies, or conduct a pilot study. However, all these methods can lead to misspecification of the NP [20-24].

Previous research has shown that RCTs in general use sample sizes that are too small due to unduly optimistic a priori assumptions [22]. This optimism is partly reflected in the assumed clinically relevant treatment effect, but can also occur as a direct effect of misspecifying a NP. For example, the value of the risk ratio (RR), which is the event rate in the experimental group divided by the event rate in the control group, is directly dependent on the event rate in the control group, which has to be estimated before the start of the trial. Similarly, for a continuous outcome, the value of the SD determines how large the difference in means is [25]. For instance, in a sample of 100 patients per arm, a difference of 10 units in some continuous measurement would be significant (*P =* 0.047) if the SD was equal to 30. If the SD was 40, this difference would no longer be statistically significant (*P* = 0.11).

**Figure 1:**
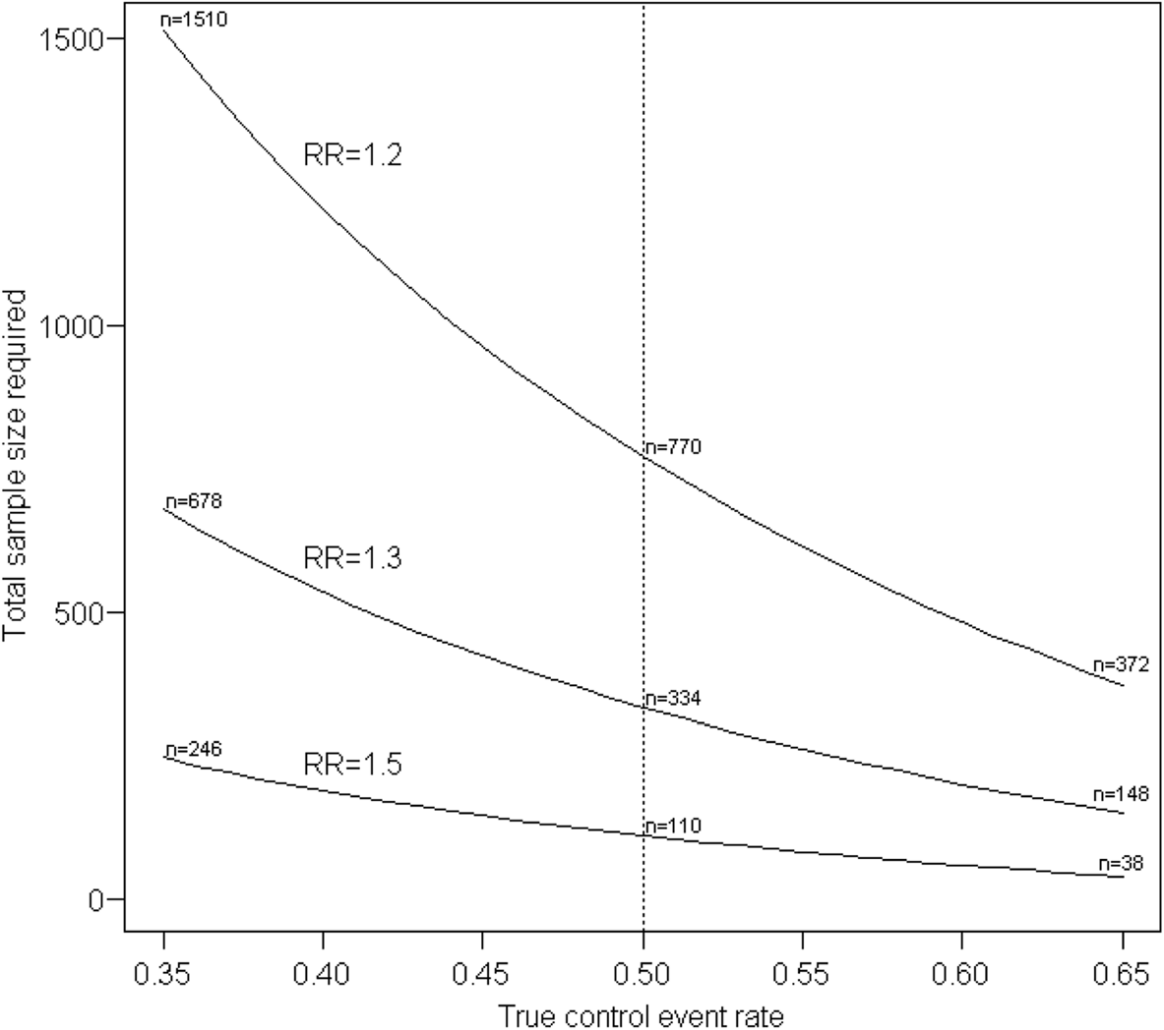
**Impact of misspecification of control event rate on total sample size.** The sample sizes required to detect RRs with 80% power at a 5% two-sided type I error level are shown. The dotted vertical line indicates the assumed CER. (See text for further explanation). CER, control group event rate; RR, risk ratio.

As an illustrative example of an RCT with a dichotomous endpoint, let us consider a clinical trial comparing the efficacy of low-dose dexamethasone with a saline placebo in ventilator-dependent infants. The clinical endpoint in this case could be survival free of major neurosensory disability after 2 years. In this case, the NP is the CER. If a CER of 50% is assumed in the design stage, a total sample size of *n* = 334 (167 per group) would be required to detect a RR of 1.3 with a 5% two-sided type I error level and 80% power. However, if the true CER is 35% instead of 50%, the required sample size to detect the same effect size, i.e. RR = 1.3, would be 678 (339 per group). Figure 1 shows how large the differences in required sample size can be for relatively small differences in the true CER, for RRs of 1.2, 1.3 and 1.5.

When comparing two groups with respect to a dichotomous outcome, the absolute risk difference is customarily used in sample size calculations as the effect size that is considered clinically relevant. The absolute risk difference is easier to interpret for clinical purposes, since it can be translated into a number needed to treat. However, for our present research we consider the RR to be a more consistent way to compare the efficacy of two treatments regardless of the CER; its value represents a relative measure of difference, taking into account the level of efficacy in the control group. For example, one could argue it is not logical to expect the same absolute difference, e.g. 20%, if the CER is 50% or 30%.

There is published research addressing the accuracy and quality of sample size calculations and their reporting in clinical trials [22,26,27]. These papers reported several discrepancies between protocols and reports [26] but also inadequate reporting and inaccuracies in general [27]. Important guidelines for the reporting of RCTs are the CONSORT statement [5] and the statement from the International Committee of Medical Journal Editors (ICMJE) on clinical trial registration [7]. Reporting of sample size calculations would be expected to have improved, since it is explicitly required by these statements.

Besides the above mentioned papers, which cover the general spectrum of sample size calculation in RCTs, little is known about the misspecification of NPs in pediatric RCTs in particular. To investigate the impact of systematic misspecification of NPs in pediatric RCTs, we reviewed published papers reporting results of pediatric RCTs. We focused on trials conducted in neonatal intensive care units and pediatric intensive care units (PICUs) due to the vulnerability of the target populations in such studies. We furthermore focused on trials evaluating pharmacological interventions because of the increased interest from regulators and the ethical considerations mentioned above. These aspects require a high standard of clinical trial design. Finally, we will provide guidance about what can be done to prevent misspecification and its consequences.

### Search strategy

We searched MEDLINE through PubMed, following the sensitivity- and precision-maximizing search strategy for identifying RCTs as suggested by the *Cochrane Handbook for Systematic Reviews of Interventions*[28]. We searched for papers between 1 January 2006 and 31 October 2011, which covers a 5-year span from the application of the clinical trial registration statement from the ICMJE. Further limits imposed were ‘Humans’ for species, ‘English’ for language and ‘All Child: 0–18 years’ for age. Additional keywords ‘Intensive care’, ‘ICU’, ‘PICU’ or ‘NICU’ were used.

### Selected articles

Selection and data extraction were performed by two authors (SN and IvdT) independently. Disagreements were discussed to reach consensus. Selection was restricted to publications concerning two-arm parallel group RCTs where efficacy assessment was the main objective. We only included trials with pharmacological interventions. Only trials with a dichotomous or a continuous outcome were included. Trials that were specifically described as Phase I or II, pilot or exploratory were excluded. We excluded trials that were designed with more than two groups (e.g. factorial designs and dose–response trials).

### Data extraction

General characteristics of each study, namely, year of publication, included patients, experimental and control interventions, primary outcome, type of primary outcome (dichotomous/continuous), registration (yes/no and if yes, registration code) and use of a crossover design were extracted. For the a priori sample size calculations, the following information was extracted: type I error, power, one- or two-sided testing, the assumed value of NPs (since we only considered dichotomous and continuous outcomes, the NPs recorded were the assumed CER and common SD, respectively), expected effect size (i.e., the standardized effect size, expressed as Cohen’s *d* for continuous outcomes, which is the mean difference between the two groups divided by the common standard deviation, and the risk ratio for dichotomous outcomes), the required sample size (with and without accounting for dropout, if applicable) and, if reported, the information source on which the assumptions concerning the NP were based, e.g. literature, own experience or pilot study. From the results sections of the articles, we extracted information on the actual sample size randomized, the one used in the analysis (irrespective of whether an intention-to-treat or per-protocol analysis was conducted), the observed value of the NP and the observed effect size.

Some papers were included in this review because the outcomes measured were continuous or dichotomous, but it was not made clear, either in the sample size calculations or in the text, which outcome was the primary one. In these cases, the primary outcome type was coded as ‘unclear’. For a trial to be considered as reporting an a priori sample size calculation, at least the power should have been mentioned in the methods section of the publication. When the type I error was not reported, a value of 0.05 (two-sided) was assumed. The reported assumed NP value was taken into consideration when it was explicitly mentioned or traceable from a cited publication; thus, we did not attempt to (re-) calculate the assumed NP from the information provided in the methods section of the article.

### Data analysis

Two authors (SN and IvdT) replicated the sample size calculations independently, based on the assumed parameters. These replicated sample sizes were calculated based on Student’s *t*-test for continuous variables and based on the chi-square test for dichotomous variables, which is equivalent to the two-sample binomial test (*Z*-test). We also recalculated required sample sizes based on the empirical values of the NPs as published in the paper. For a continuous outcome for which median and range were reported instead of mean and SD, the SD was calculated according to Hozo *et al*. [29].

**Figure 2:**
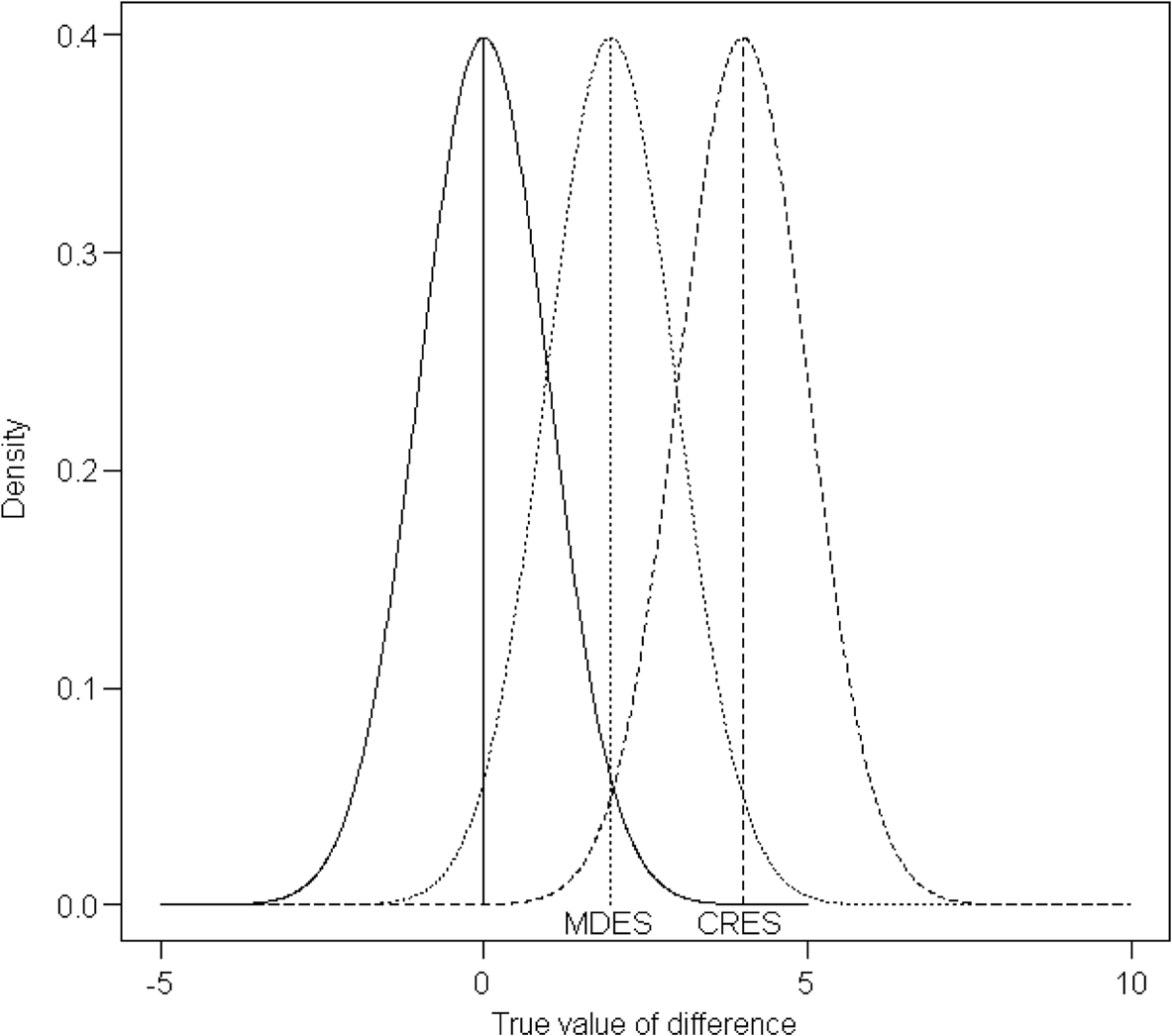
**Illustration of minimum detectable effect size.** The minimum detectable effect size (MDES) is the minimum difference between groups that yields a statistically significant result. The power of the study is calculated using the clinically relevant effect size (CRES). The density of the left curve to the right of the MDES represents the type I error, i.e. 2.5% (one-sided) in this case. The density of the middle curve to the right of the MDES represents the power for the MDES to be detected, i.e. 50%. The density of the right curve to the right of the MDES represents the power for the CRES to be detected, i.e. the power of such a study, which is 98% in this example. CRES, clinically relevant effect size; MDES, minimum detectable effect size.

We also calculated the minimum detectable effect size (MDES), given the sample size available for analysis and the observed NP (Figure 2). Note that this quantity is different from the effect size expected for the a priori sample size calculation. MDES reflects the minimum difference that would yield a significant result for a given sample size, NP and type I error level; this effect size will be smaller than the effect size on which the sample size calculation is based, i.e. the clinically relevant effect size. MDES has a power of 50% to be detected if it is true, therefore for any trial designed with more than 50% power for a clinically relevant effect size, the MDES will be smaller than the latter [30].

The per trial estimate of misspecification of the NP was calculated relative to the observed NP as:

$$\left(\mathrm{Assumed}\;\mathrm{NP}\;–\;\mathrm{Observed}\;\mathrm{NP}\right)\;/\;\mathrm{Observed}\;\mathrm{NP}.$$

The relative difference for the MDES was calculated in the same way:

$$\left(\mathrm{MDES}\;\mathrm{with}\;\mathrm{the}\;\mathrm{assumed}\;\mathrm{NP}\;–\;\mathrm{MDES}\;\mathrm{with}\;\mathrm{the}\;\mathrm{observed}\;\mathrm{NP}\right)\;/\;\mathrm{MDES}\;\mathrm{with}\;\mathrm{the}\;\mathrm{observed}\;\mathrm{NP}.$$

As mentioned before, empirically obtained estimators of nuisance parameters are also subject to random variation; therefore any systematic trend in the direction of possible misspecifications was our main interest. Statistical analyses were conducted with R, version 2.13.1 and SPSS (PASW statistics) version 17.

### Results

**Figure 3:**
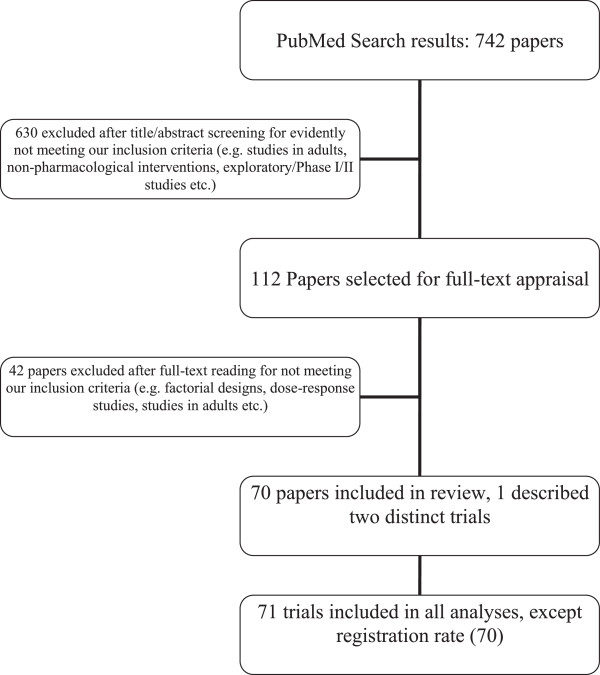
Flowchart of the search and inclusion procedure.

The search yielded 742 citations. After screening titles and abstracts, 112 articles were selected for full-text appraisal. Of these 112 articles, 70 complied with our inclusion criteria. One of the trials reported two distinct subgroups for which a different power calculation and hypothesis were described, and they were taken into account as two distinct trials for all the analyses except the registration rate, leading to a total of 71 trials described in 70 papers (see Figure 3). One trial had a crossover design with a dichotomous outcome and used the McNemar test for sample size calculation as well as for analysis. Therefore, this study was included in the analysis concerning misspecification of NPs but not in recalculation of sample and effect sizes, because even though the NP of the McNemar test is still a proportion (of discordant pairs), the effect size is not comparable with the one in a chi-square test.

The PRISMA checklist can be found in Additional file 1, the reviewed articles in Additional file 2 and the extracted data in Additional file 3.

#### *Descriptive characteristics*

Table 1 summarizes the basic characteristics of the included trials. Of the 71 trials, 31 were performed for preterm or term neonates. The information concerning the sample size calculations is shown in Table 2. Of the 12 trials reporting on-line registration, 3 (25%) were registered in the International Standard Randomized Controlled Trial Number Register (ISRCTN) and 9 (75%) were registered in ClinicalTrials.gov (NCT). In 11 of the 49 papers (22%) that reported a sample size calculation in the methods section, a different analysis was used to evaluate the result of the trial than the one assumed in the sample size calculation. Four of these applied nonparametric analyses, presumably due to a failure to meet the assumptions of the parametric tests and/or unfamiliarity with sample size calculations for non-normally distributed outcomes.

**Table 1:** Basic characteristics of the 70 included papers

**Characteristic**	***N *** **(%)**
Registration	
Registration reported	12 (17)
Study population	
Neonates (0 to 1 years old)	31 (44)
Children (>1 years old)	24 (34)
Both	15 (21)
Intervention in the control group	
Placebo	30 (43)
Active control	35 (50)
Standard care/none	5 (7)
Funding source	
Public	19 (27)
Private	6 (9)
Not clear	45 (64)

**Table 2:** Characteristics of sample size calculations of the 71 included trials (70 papers)

**Characteristic**	***N *** **(%)**
Type of primary outcome	
Dichotomous	30 (42)
Continuous	39 (55)
Unclear	2 (3)
A priori sample size calculation	
At least power reported	49 (69)
→ Of these	
NP reported	35 (71)
NP reported as proportion of total	35 (49)
No report of a priori sample size calculation	22 (31)
Information source of NP assumption	
→ Of the 49 trials that report at least power	
Literature	21 (43)
Own experience	9 (18)
Pilot study	6 (12)
Not reported	13 (27)
→ Of the 35 trials that report an assumed NP value	
Literature	14 (40)
Own experience	8 (23)
Pilot study	6 (17)
Not reported	7 (20)

In the reports of all 12 registered trials, an a priori sample size calculation (at least power mentioned) was reported; this was the case in 35 reports out of 58 trials (60%) that did not report a registration. The rate of reporting the expected NP was 10 out of 12 for papers that reported registration (83%) and 24 out of 58 (41%) for papers that did not. Note that for these figures the number of papers is the total sample size (70) rather than the number of trials (71).

#### *Misspecification of nuisance parameters*

**Figure 4:**
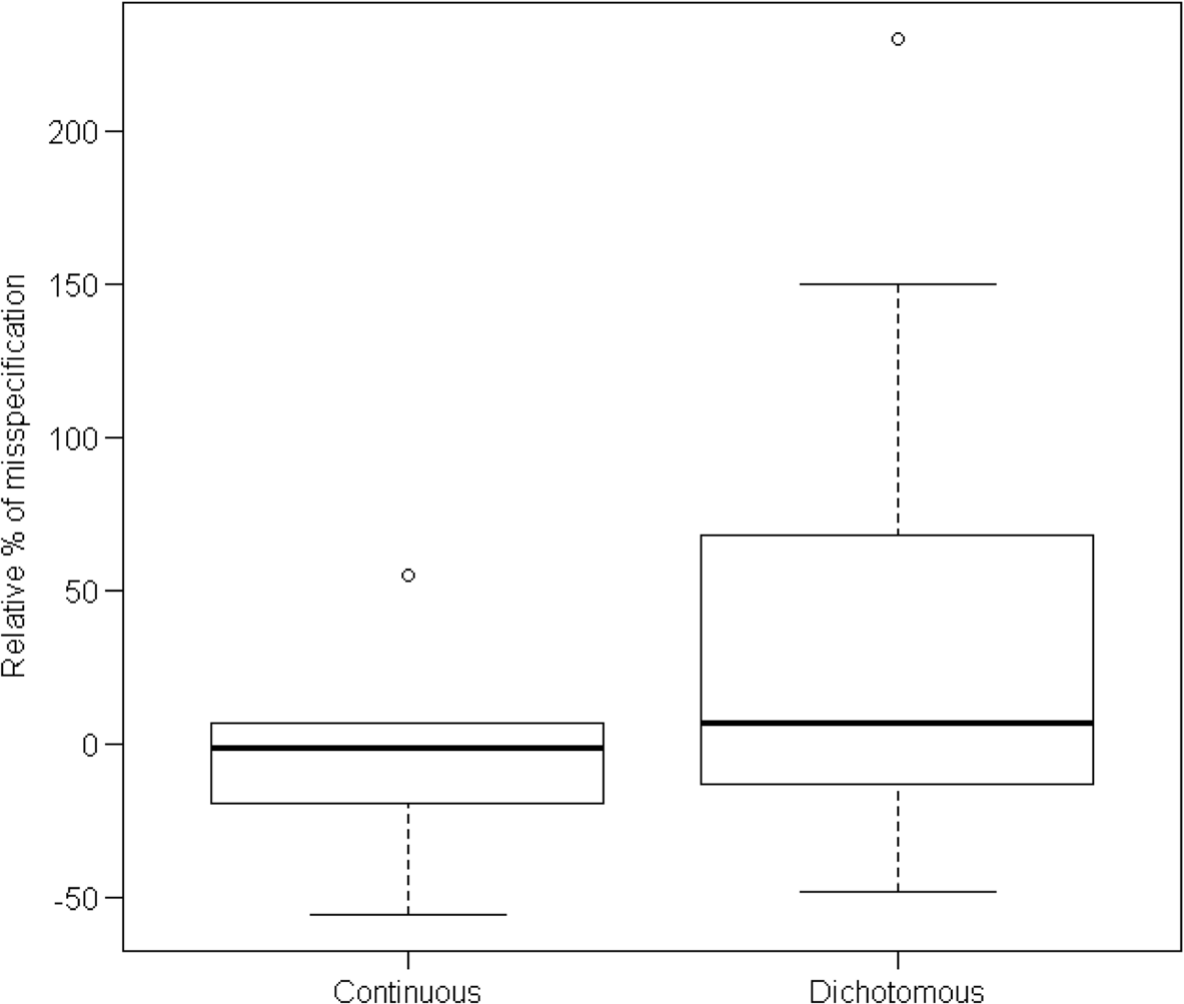
**Misspecification of nuisance parameters.** Relative misspecification of NPs in the trials reviewed = (Expected value of NP – Observed value of NP)/Observed value of NP.

Seven of the 35 articles report an assumed value for the NP, but did not report the observed value for this parameter in the results section, because a different analysis was conducted (e.g. nonparametric testing). Thus, we were able to calculate the relative misspecification for 28 trials, 22 with a dichotomous and 6 with a continuous primary outcome. The results are summarized in Figure 4. As can be seen, CERs were more often overestimated than underestimated; SDs were more often underestimated. The median [inter-quartile range (IQR)] overestimation was 6.8 [-12.1, 57.8]% for trials with dichotomous outcomes and -1.5 [-15.3, 5.1]% for trials with continuous outcomes. As shown in the example above, overestimation of the control event rate can have severe consequences for the sample size requirements. It is indicative that the total estimated sample size for the subgroup of trials with continuous outcomes was 846 subjects though it should have been 1,044 subjects, while for the ones with dichotomous outcomes, in total 3,326 subjects were planned to be enrolled, while 3,904 should have been enrolled based on the values for the NP actually observed.

The effect of the misspecification of the NP was apparent on the average power of the studies reviewed. The average power required by design was 83% while the average power taking the observed NPs into account, based on the sample sizes calculated in the papers, would have been 73.9%. Based on our replicated sample sizes the power achieved would be 71.8%. However, these results should only be taken as indicative and exploratory, as we share the concerns of other authors about power calculations after data is collected [31]. More specifically, researchers should be very careful with interpreting the post-hoc power, which is the power calculated for the observed treatment effect, and the same applies to the observed NP. There was no evidence of a relation between the source used to make assumptions for the NP and the magnitude of misspecification.

#### *Minimum detectable effect size in trials with dichotomous outcomes*

**Figure 5:**
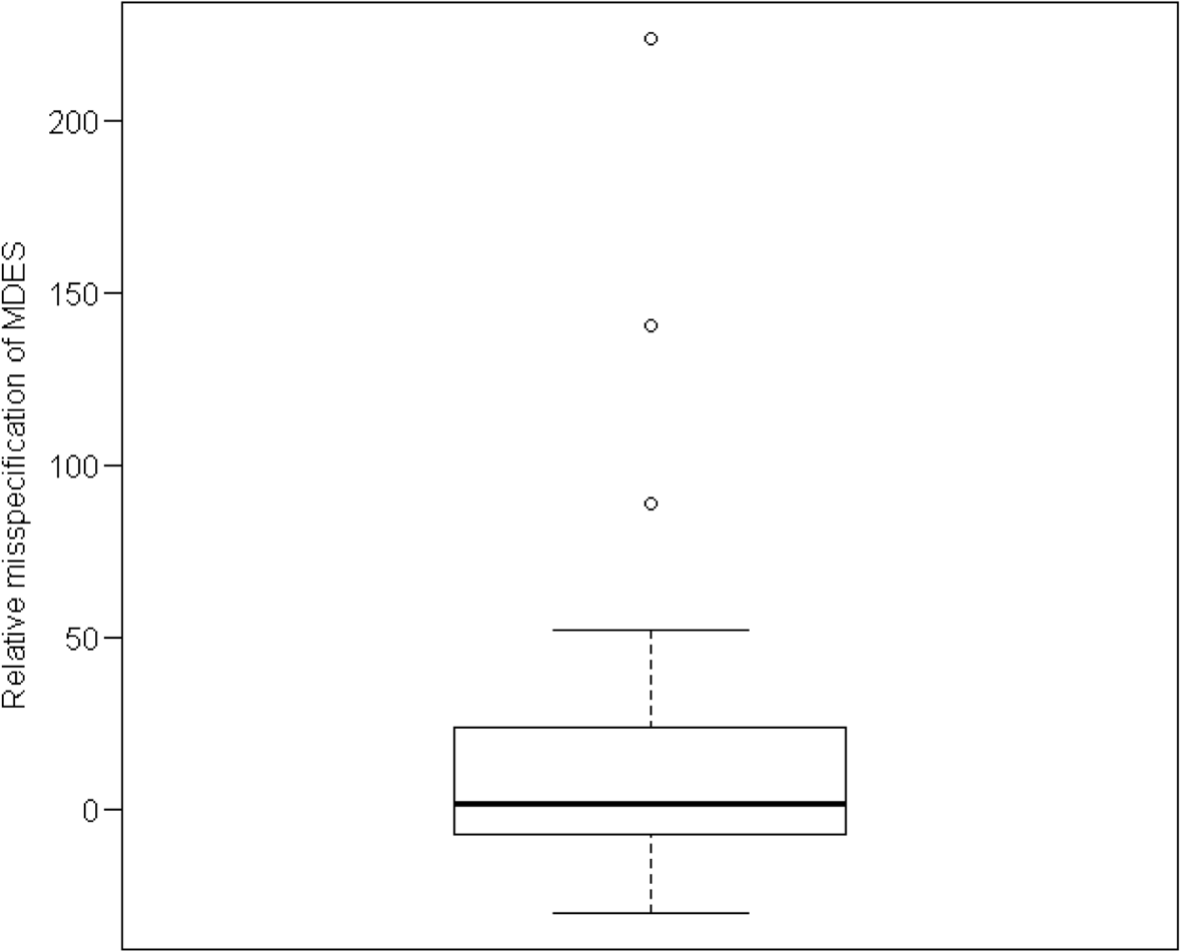
**Relative misspecification (as a percentage) of MDES in trials with a dichotomous outcome.** The vertical axis represents the quantity: (MDES with the assumed NP – MDES with the observed NP)/MDES with the observed NP. MDES, minimum detectable effect size; NP, nuisance parameter.

Relative differences between the MDES based on a priori assumptions and the MDES based on the observed values of the NPs are presented in Figure 5. The proportion of trials in which the observed MDES was larger than the assumed MDES was roughly equal to the proportion of trials in which it was smaller (the median misspecification was 1%). However, the distribution of the discrepancies is skewed (minimum = -30%, maximum = 224%, IQR = -7% to 23%), with some extreme values, reflecting the skewed distribution of the discrepancies in the NPs. This shows that the misspecification of NP more often causes MDES to be smaller than anticipated by design, therefore making significant findings less probable.

### Discussion

In this review of 71 pediatric clinical trials, our main goal was to assess the presence and magnitude of systematic misspecification of NPs in sample size calculations. Deviations between assumed and realized values of NPs can lead to undesirable trial characteristics like underestimated sample size and overestimated power. This can possibly lead to important clinical improvements being missed and to an increased number of trials unnecessarily considered negative or failures. It also reduces the value of individual patients participating in clinical trials. Some experts consider underpowered trials to be unethical [32].

Of course, observed parameter values deviate from the assumed ones, due to random fluctuation and this is incorporated in sample size estimation. If estimation is accurate, it is expected that these discrepancies will take place in both directions (both over- and underestimating), causing no overall effect in the design characteristics of the RCTs reviewed. However, as the results of our review show, there is systematic misspecification of nuisance parameters, resulting in about 10% lower average power of the studies than required in the design stage. As a result, more patients should have been studied for the conclusions of the studies to be in compliance with their design characteristics. The loss in power theoretically results in 10% of studies with promising interventions being expected to conclude incorrectly that there is no benefit.

An important issue of concern is that reporting of sample size calculations is still not adequate. This is in accordance with the findings by Charles *et al*. [27]. We assumed that the CONSORT statement and the clinical trials registration would have led to more transparent reporting, but the percentage of registered trials was very low (17%). It should be noted though that while trial registration was stated as a requirement for publication by ICMJE, we did not restrict our search to these journals. The rate of registration may in reality have been higher, since our information depended on explicit reporting in journal articles.

Misspecification of the NP has more severe consequences for trials with a dichotomous outcome than for those with a continuous outcome. As the results of our review show, the CER was found to be up to 200% misspecified. One way around this is to avoid dichotomizing continuous outcomes, if possible, and also to avoid treating time-to-event outcomes as binary. Misspecification, especially underestimation, of the SD for a trial with a continuous outcome also has considerable consequences. We are unable to draw reliable conclusions from our study, because of the very small number of trials with a continuous endpoint reporting both assumed and realized values of the standard deviation.

Further limitations of our study are the quite specific inclusion criteria (trials with pharmacological interventions and conducted in an ICU). The findings may not be generalizable beyond this group of trials. Additionally, RCTs that are not analyzed by the intention-to-treat principle are likely to introduce bias in estimation of the treatment effect, which could also have implications for sample size calculations. However, it was seldom reported whether the trial was analyzed by the intention-to-treat principle (in only 19 papers was it clearly stated). Furthermore, even though the search was conducted in a systematic way, the possibility that some trials that could fit our inclusion criteria were missed, cannot be excluded. However, we do not expect this to affect the validity of our results since the scope of this review is to explore the state of affairs rather than, e.g., evaluate the effectiveness of an intervention where missing a trial would be considered a caveat.

Misspecification of NPs occurs frequently in pediatric clinical trials conducted in ICUs. Failure of reporting a priori assumptions about NPs appeared to be more common in the trial reports included in this review than in trials published in high-impact medical journals [27], even though these trials included an extremely vulnerable population. Awareness should be raised of this matter and journal editors should be more demanding concerning reporting standards adopted by the high-impact journals.

Methodologies exist that are less sensitive to assumptions of NPs, such as using a more flexible design and analysis (e.g. sequential trials) or re-estimation of the sample size (internal pilot). Another way would be to state the expectations for the clinically relevant effect size in a standardized way (e.g. use of Cohen’s standardized effect size, [25,33]). This allows one not to make specific assumptions for the NP but rather state the magnitude of the effect size considered clinically relevant (e.g. small, medium or large effect size).

## Conclusions

Research in vulnerable populations, like children, is challenging and demanding. Cumulative knowledge is difficult to acquire but necessary for evidence-based evaluation of medical interventions. This should be done in the most efficient and ethical way possible and a well-thought-out study design is a crucial step towards this goal. We would strongly advise editors of all medical journals to adopt the reporting standards guidance and be more demanding that authors conform to these standards.

## Abbreviations

CER: control group event rate; CRES: clinically relevant effect size; ICMJE: International Committee of Medical Journal Editors; IQR: inter-quartile range; MDES: minimum detectable effect size; NP: nuisance parameter; PICU: pediatric intensive care unit; RCT: randomized controlled trial; RR: risk ratio; SD: standard deviation.

## Competing interests

The authors declare that they have no competing interests.

## Authors’ contributions

SN conducted the literature search, study selection and data extraction; carried out the analyses; drafted and revised the manuscript; and approved the final manuscript as submitted. KCBR conceptualized the study, reviewed the manuscript and approved the final manuscript as submitted. JHvdL reviewed the manuscript, extracted relevant medical information and approved the final manuscript as submitted. IvdT conceptualized the study, selected the studies and extracted data, reviewed the analyses, reviewed the manuscript and approved the final manuscript as submitted.

## Supplementary Material

Additional file 1 PRISMA checklist of the systematic review. Click here for file

Additional file 2 Articles reviewed. Click here for file

Additional file 3 Data extracted from the reviewed articles. Click here for file
