# Supplementary material for: Sample size calculations in pediatric clinical trials conducted in an ICU: a systematic review
Source: Trials. 2014 Jul 8;15:274. doi: 10.1186/1745-6215-15-274 (PMC4107993; doi:10.1186/1745-6215-15-274)
Supplement: Additional file 2 — Articles reviewed. [file 1745-6215-15-274-S2.pdf]

Reviewed Papers

(1) Almenrader N, Passariello M, Coccetti B, Haiberger R, Pietropaoli P. Premedication in children: a comparison of oral midazolam and oral clonidine. Paediatr Anaesth 2007 Dec;17(12):1143-9.

(2) Arnon S.S., Schechter R. , Maslanka, S.E., Jewell, N.P., Hatheway, C.L. Human botulism immune globulin for the treatment of infant botulism. N. Engl. J. Med. 2006; 354 (5) : 462 - 471.

(3) Armangil,D., Yurdakok,M., Korkmaz,A., Yigit,S., Tekinalp,G. Inhaled beta-2 agonist salbutamol for the treatment of transient tachypnea of the newborn J. Pediatr. 2011; 159 (3):398-403.

(4) Beardsall K, Vanhaesebrouck S, Ogilvy-Stuart AL, Vanhole C, Palmer CR, van WM, et al. Early insulin therapy in very-low-birth-weight infants. N Engl J Med 2008 Oct 30;359(18):1873-84.

(5) Ben KS, Blidi S, Trifa M, Skhiri A, Drira M, Regaya T, et al. Time to extubation in infants undergoing pyloromyotomy -- isoflurane inhalation vs remifentanil infusion. Middle East J Anesthesiol 2009 Jun;20(2):277-80.

(6) Bigham MT, Jacobs BR, Monaco MA, Brilli RJ, Wells D, Conway EM, et al. Helium/oxygen-driven albuterol nebulization in the management of children with status asthmaticus: a randomized, placebo-controlled trial. Pediatr Crit Care Med 2010 May;11(3):356-61.

(7) Bogie AL, Towne D, Luckett PM, Abramo TJ, Wiebe RA. Comparison of intravenous terbutaline versus normal saline in pediatric patients on continuous high-dose nebulized albuterol for status asthmaticus. Pediatr Emerg Care 2007 Jun;23(6):355-61.

(8) Bonsante F, Latorre G, Iacobelli S, Forziati V, Laforgia N, Esposito L, et al. Early low-dose hydrocortisone in very preterm infants: a randomized, placebo-controlled trial. Neonatology 2007;91(4):217-21.

(9) Boogaard R, Hulsmann AR, van VL, Vaessen-Verberne AA, Yap YN, Sprij AJ, et al. Recombinant human deoxyribonuclease in infants with respiratory syncytial virus bronchiolitis. Chest 2007 Mar;131(3):788-95.

(10) Boyer LV, Theodorou AA, Berg RA, Mallie J, Chavez-Mendez A, Garcia-Ubbelohde W, et al. Antivenom for critically ill children with neurotoxicity from scorpion stings. N Engl J Med 2009 May 14;360(20):2090-8.

(11) Cattarelli D, Spandrio M, Gasparoni A, Bottino R, Offer C, Chirico G. A randomised, double blind, placebo controlled trial of the effect of theophylline in prevention of vasomotor nephropathy in very preterm neonates with respiratory distress syndrome. Arch Dis Child Fetal Neonatal Ed 2006 Mar;91(2):F80-F84.

(12) Chhabra A, Sinha R, Subramaniam R, Chandra P, Narang D, Garg SP. Comparison of sub-Tenon's block with i.v. fentanyl for paediatric vitreoretinal surgery. Br J Anaesth 2009 Nov;103(5):739-43.

(13) Choong K, Alfaleh K, Doucette J, Gray S, Rich B, Verhey L, et al. Remifentanil for endotracheal intubation in neonates: a randomised controlled trial. Arch Dis Child Fetal Neonatal Ed 2010 Mar;95(2):F80-F84.

(14) Chu YC, Lin SM, Hsieh YC, Chan KH, Tsou MY. Intraoperative administration of tramadol for postoperative nurse-controlled analgesia resulted in earlier awakening and less sedation than morphine in children after cardiac surgery. Anesth Analg 2006 Jun;102(6):1668-73.

(15) Cignacco E, Hamers JP, van Lingen RA, Zimmermann LJ, Muller R, Gessler P, et al. Pain relief in ventilated preterms during endotracheal suctioning: a randomized controlled trial. Swiss Med Wkly 2008 Nov 1;138(43-44):635-45.

(16) Cogen MS, Parker JS, Sleep TE, Elsas FJ, Metz TH, Jr., McGwin G, Jr. Masked trial of topical anesthesia for retinopathy of prematurity eye examinations. J AAPOS 2011 Feb;15(1):45-8.

(17) Cresi F, Marinaccio C, Russo MC, Miniero R, Silvestro L. Short-term effect of domperidone on gastroesophageal reflux in newborns assessed by combined intraluminal impedance and pH monitoring. J Perinatol 2008 Nov;28(11):766-70.

(18) Darnell CM, Thompson J, Stromberg D, Roy L, Sheeran P. Effect of low-dose naloxone infusion on fentanyl requirements in critically ill children. Pediatrics 2008 May;121(5):e1363-e1371.

(19) Doyle LW, Davis PG, Morley CJ, McPhee A, Carlin JB. Outcome at 2 years of age of infants from the DART study: a multicenter, international, randomized, controlled trial of low-dose dexamethasone. Pediatrics 2007 Apr;119(4):716-21.

(20) Duman A, Apiliogullari S, Duman I. Effects of intrathecal fentanyl on quality of spinal anesthesia in children undergoing inguinal hernia repair. Paediatr Anaesth 2010 Jun;20(6):530-6.

(21) Erol A, Tavlan A, Tuncer S, Topal A, Yurtcu M, Reisli R, et al. Caudal anesthesia for minor subumbilical pediatric surgery: a comparison of levobupivacaine alone and levobupivacaine plus sufentanil. J Clin Anesth 2008 Sep;20(6):442-6.

(22) Eslami Z, Shajari A, Kheirandish M, Heidary A. Theophylline for prevention of kidney dysfunction in neonates with severe asphyxia. Iran J Kidney Dis 2009 Oct;3(4):222-6.

(23) Ferreira CA, Vicente WV, Evora PR, Rodrigues AJ, Klamt JG, Carlotti AP, et al. High-dose aprotinin does not affect troponin I, N-Terminal pro-B-type natriuretic peptid and renal function in children submitted to surgical correction with extracorporeal circulation. Rev Bras Cir Cardiovasc 2009 Dec;24(4):519-32.

(24) Fujii AM, Patel SM, Allen R, Doros G, Guo CY, Testa S. Poractant alfa and beractant treatment of very premature infants with respiratory distress syndrome. J Perinatol 2010 Oct;30(10):665-70.

(25) Galante D, Pellico G, Meola S, Caso A, De CA, Milillo R, et al. Hemodynamic effects of levobupivacaine after pediatric caudal anesthesia evaluated by transesophageal doppler. Paediatr Anaesth 2008 Nov;18(11):1066-74.

(26) Ghai B, Ram J, Makkar JK, Wig J, Kaushik S. Subtenon block compared to intravenous fentanyl for perioperative analgesia in pediatric cataract surgery. Anesth Analg 2009 Apr;108(4):1132-8.

(27) Ghai B, Makkar JK, Chari P, Rao KL. Addition of midazolam to continuous postoperative epidural bupivacaine infusion reduces requirement for rescue analgesia in children undergoing upper abdominal and flank surgery. J Clin Anesth 2009 Mar;21(2):113-9.

(28) Gounaris A, Costalos C, Varchalama E, Kokori F, Grivea IN, Konstantinidi K, et al. Gastric emptying of preterm neonates receiving domperidone. Neonatology 2010;97(1):56-60.

(29) Gutmann A, Pessenbacher K, Gschanes A, Eggenreich U, Wargenau M, Toller W. Propofol anesthesia in spontaneously breathing children undergoing magnetic resonance imaging: comparison of two propofol emulsions. Paediatr Anaesth 2006 Mar;16(3):266-74.

(30) Hassid S, Nicaise C, Michel F, Vialet R, Thomachot L, Lagier P, et al. Randomized controlled trial of sevoflurane for intubation in neonates. Paediatr Anaesth 2007 Nov;17(11):1053-8.

(31) Jacomo AD, Carmona F, Matsuno AK, Manso PH, Carlotti AP. Effect of oral hygiene with 0.12% chlorhexidine gluconate on the incidence of nosocomial pneumonia in children undergoing cardiac surgery. Infect Control Hosp Epidemiol 2011 Jun;32(6):591-6.

(32) Jeschke MG, Finnerty CC, Suman OE, Kulp G, Mlcak RP, Herndon DN. The effect of oxandrolone on the endocrinologic, inflammatory, and hypermetabolic responses during the acute phase postburn. Ann Surg 2007 Sep;246(3):351-60.

(33) Kaabachi O, Zarghouni A, Ouezini R, Abdelaziz AB, Chattaoui O, Kokki H. Clonidine 1 microg/kg is a safe and effective adjuvant to plain bupivacaine in spinal anesthesia in adolescents. Anesth Analg 2007 Aug;105(2):516-9.

(34) Kaabachi O, Chettaoui O, Ouezini R, Abdelaziz AB, Cherif R, Kokki H. A ketamine-propofol admixture does not reduce the pain on injection compared with a lidocaine-propofol admixture. Paediatr Anaesth 2007 Aug;17(8):734-7.

(35) Karanovic N, Carev M, Ujevic A, Kardum G, Dogas Z. Association of oculocardiac reflex and postoperative nausea and vomiting in strabismus surgery in children anesthetized with halothane and nitrous oxide. Paediatr Anaesth 2006 Sep;16(9):948-54.

(36) Kneyber MC, van Woensel JB, Uijtendaal E, Uiterwaal CS, Kimpen JL. Azithromycin does not improve disease course in hospitalized infants with respiratory syncytial virus (RSV) lower respiratory tract disease: a randomized equivalence trial. Pediatr Pulmonol 2008 Feb;43(2):142-9.

(37) Koruk S, Mizrak A, Kaya UB, Ilhan O, Baspinar O, Oner U. Propofol/dexmedetomidine and propofol/ketamine combinations for anesthesia in pediatric patients undergoing transcatheter atrial septal defect closure: a prospective randomized study. Clin Ther 2010 Apr;32(4):701-9.

(38) Kuhn P, Messer J, Paupe A, Espagne S, Kacet N, Mouchnino G, et al. A multicenter, randomized, placebo-controlled trial of prophylactic recombinant granulocyte-colony stimulating factor in preterm neonates with neutropenia. J Pediatr 2009 Sep;155(3):324-30.

(39) Lago P, Tiozzo C, Boccuzzo G, Allegro A, Zacchello F. Remifentanil for percutaneous intravenous central catheter placement in preterm infant: a randomized controlled trial. Paediatr Anaesth 2008 Aug;18(8):736-44.

(40) Lee BS, Byun SY, Chung ML, Chang JY, Kim HY, Kim EA, et al. Effect of furosemide on ductal closure and renal function in indomethacin-treated preterm infants during the early neonatal period. Neonatology 2010;98(2):191-9.

(41) Lemyre B, Hogan DL, Gaboury I, Sherlock R, Blanchard C, Moher D. How effective is tetracaine 4% gel, before a venipuncture, in reducing procedural pain in infants: a randomized double-blind placebo controlled trial. BMC Pediatr 2007;7:7.

(42) Lucas da Silva PS, Oliveira Iglesias SB, Leao FV, Aguiar VE, Brunow de CW. Procedural sedation for insertion of central venous catheters in children: comparison of midazolam/fentanyl with midazolam/ketamine. Paediatr Anaesth 2007 Apr;17(4):358-63.

(43) Marraro GA, Luchetti M, Spada C, Galassini E, Giossi M, Piero AM. Selective medicated (normal saline and exogenous surfactant) bronchoalveolar lavage in severe aspiration syndrome in children. Pediatr Crit Care Med 2007 Sep;8(5):476-81.

(44) Michel F, Vialet R, Hassid S, Nicaise C, Garbi A, Thomachot L, et al. Sevoflurane for central catheter placement in neonatal intensive care: a randomized trial. Paediatr Anaesth 2010 Aug;20(8):712-9.

(45) Murugesan C, Banakal SK, Garg R, Keshavamurthy S, Muralidhar K. The efficacy of aprotinin in arterial switch operations in infants. Anesth Analg 2008 Sep;107(3):783-7.

(46) Nagdeve NG, Yaddanapudi S, Pandav SS. The effect of different doses of ketamine on intraocular pressure in anesthetized children. J Pediatr Ophthalmol Strabismus 2006 Jul;43(4):219-23.

(47) Namachivayam P, Theilen U, Butt WW, Cooper SM, Penny DJ, Shekerdemian LS. Sildenafil prevents rebound pulmonary hypertension after withdrawal of nitric oxide in children. Am J Respir Crit Care Med 2006 Nov 1;174(9):1042-7.

(48) Nyman Y, von HK, Palm C, Eksborg S, Lonnqvist PA. Etomidate-Lipuro is associated with considerably less injection pain in children compared with propofol with added lidocaine. Br J Anaesth 2006 Oct;97(4):536-9.

(49) Nyman Y, von HK, Ritzmo C, Eksborg S, Lonnqvist PA. Effect of a small priming dose on myoclonic movements after intravenous anaesthesia induction with Etomidate-Lipuro in children. Br J Anaesth 2011 Aug;107(2):225-8.

(50) Parikh TB, Nanavati RN, Patankar CV, Rao S, Bisure K, Udani RH, et al. Fluconazole prophylaxis against fungal colonization and invasive fungal infection in very low birth weight infants. Indian Pediatr 2007 Nov;44(11):830-7.

(51) Pasha YZ, hmadpour-Kacho M, Hajiahmadi M, Hosseini MB. Enteral erythropoietin increases plasma erythropoietin level in preterm infants: a randomized controlled trial. Indian Pediatr 2008 Jan;45(1):25-8.

(52) Pedreira ML, Kusahara DM, de Carvalho WB, Nunez SC, Peterlini MA. Oral care interventions and oropharyngeal colonization in children receiving mechanical ventilation. Am J Crit Care 2009 Jul;18(4):319-28.

(53) Penido MG, de Oliveira Silva DF, Tavares EC, Silva YP. Propofol versus midazolam for intubating preterm neonates: a randomized controlled trial. J Perinatol 2011 May;31(5):356-60.

(54) Pourarian S, Pishva N, Madani A, Rastegari M. Comparison of oral ibuprofen and indomethacin on closure of patent ductus arteriosus in preterm infants. East Mediterr Health J 2008 Mar;14(2):360-5.

(55) Riethmueller J, Borth-Bruhns T, Kumpf M, Vonthein R, Wiskirchen J, Stern M, et al. Recombinant human deoxyribonuclease shortens ventilation time in young, mechanically ventilated children. Pediatr Pulmonol 2006 Jan;41(1):61-6.

(56) Santos AR, Heidemann SM, Walters HL, III, Delius RE. Effect of inhaled corticosteroid on pulmonary injury and inflammatory mediator production after cardiopulmonary bypass in children. Pediatr Crit Care Med 2007 Sep;8(5):465-9.

(57) Schroeder AR, Axelrod DM, Silverman NH, Rubesova E, Merkel E, Roth SJ. A continuous heparin infusion does not prevent catheter-related thrombosis in infants after cardiac surgery. Pediatr Crit Care Med 2010 Jul;11(4):489-95.

(58) Shi Y, Zhao J, Tang S, Pan F, Liu L, Tian Z, et al. Effect of hemocoagulase for prevention of pulmonary hemorrhage in critical newborns on mechanical ventilation: a randomized controlled trial. Indian Pediatr 2008 Mar;45(3):199-202.

(59) Simons SH, Roofthooft DW, Van DM, van Lingen RA, Duivenvoorden HJ, van den Anker JN, et al. Morphine in ventilated neonates: its effects on arterial blood pressure. Arch Dis Child Fetal Neonatal Ed 2006 Jan;91(1):F46-F51.

(60) Singh R, Batra YK, Bharti N, Panda NB. Comparison of propofol versus propofol-ketamine combination for sedation during spinal anesthesia in children: randomized clinical trial of efficacy and safety. Paediatr Anaesth 2010 May;20(5):439-44.

(61) Sinha A, Jayashree M, Singhi S. Aerosolized L-epinephrine vs budesonide for post extubation stridor: a randomized controlled trial. Indian Pediatr 2010 Apr;47(4):317-22.

(62) Soltesz S, Silomon M, Graf G, Mencke T, Boulaadass S, Molter GP. Effect of a 0.5% dilution of propofol on pain on injection during induction of anesthesia in children. Anesthesiology 2007 Jan;106(1):80-4.

(63) Sorce LR, Hamilton SM, Gauvreau K, Mets MB, Hunter DG, Rahmani B, et al. Preventing corneal abrasions in critically ill children receiving neuromuscular blockade: a randomized, controlled trial. Pediatr Crit Care Med 2009 Mar;10(2):171-5.

(64) Uslu S, Ozdemir H, Comert S, Bolat F, Nuhoglu A. The effect of low-dose heparin on maintaining peripherally inserted percutaneous central venous catheters in neonates. J Perinatol 2010 Dec;30(12):794-9.

(65) van Woensel JB, Vyas H. Dexamethasone in children mechanically ventilated for lower respiratory tract infection caused by respiratory syncytial virus: a randomized controlled trial. Crit Care Med 2011 Jul;39(7):1779-83.

(66) Vargas-Origel A, Gomez-Rodriguez G, na-Valenzuela C, Vela-Huerta MM, arcon-Santos SB, mador-Licona N. The use of sildenafil in persistent pulmonary hypertension of the newborn. Am J Perinatol 2010 Mar;27(3):225-30.

(67) Vlasselaers D, Milants I, Desmet L, Wouters PJ, Vanhorebeek I, van dH, I, et al. Intensive insulin therapy for patients in paediatric intensive care: a prospective, randomised controlled study. Lancet 2009 Feb 14;373(9663):547-56.

(68) Williams GD, Ramamoorthy C, Pentcheva K, Boltz MG, Kamra K, Reddy VM. A randomized, controlled trial of aprotinin in neonates undergoing open-heart surgery. Paediatr Anaesth 2008 Sep;18(9):812-9.

(69) Xia WF, Liu Y, Zhou QS, Tang QZ, Zou HD. Comparison of the effects of propofol and midazolam on inflammation and oxidase stress in children with congenital heart disease undergoing cardiac surgery. Yonsei Med J 2011 Mar;52(2):326-32.

(70) Yildizdas D, Yapicioglu H, Celik U, Sertdemir Y, Alhan E. Terlipressin as a rescue therapy for catecholamine-resistant septic shock in children. Intensive Care Med 2008 Mar;34(3):511-7.
