# Supplementary material for: Sample size calculations in pediatric clinical trials conducted in an ICU: a systematic review
Source: Trials. 2014 Jul 8;15:274. doi: 10.1186/1745-6215-15-274 (PMC4107993; doi:10.1186/1745-6215-15-274)
Supplement: Additional file 3 — Data extracted from the reviewed articles. [file 1745-6215-15-274-S3.pdf]

Paper	Year of Publication	Registration Number	Outcome (Continuous/Dichotomous)	Crossover Design (Yes/No)	Type I Error by Design (reported in the Methods section)	Testing (One/Two Sided, NR: Not reported)	Type II Error (1-power) by Design (reported in the Methods section)	Expected Nuisance Parameter (Reported in the Methods Section) - Standard deviation for continuous outcomes, control group event rate for dichotomous outcomes)	Total Sample Size by Design (reported in the Methods section)	Source of information for nuisance parameter estimation (Lit=Literature, OE = Own Experience, PS= Pilot study)	Expected Effect Size (cohen's d for continuous outcomes, risk ratio for dichotomous outcomes)	Observed Nuisance Parameter (from Results Section)
Almenrader et al, 2007	2007	NR	Dichotomous	NO	0.05	NR	0.2	0.79	60	Lit	1.25	0.76
Armangil et al, 2011	2011	NR	Continuous	NO	0.05	NR	NR	NR	NR	NR	NR	NR
Arnon et al, 2006	2006	NR	Continuous	NO	0.05	NR	0.2	NR	120	NR	NR	4.17
Beardsall et al, 2008	2008	ISRCTN78428828	Dichotomous	NO	0.05	NR	0.2	0.2	430	PS	0.5	0.09
Bigham et al, 2010	2010	NCT00410150	Continuous	NO	0.05	two-sided	0.2	1.6	348	OE	-0.31	1.65
Bogie et al, 2007	2007	NR	Continuous	NO	0.05	one-sided	0.18	1.5	60	NR	-0.67	3.4
Bonsante et al, 2007	2007	NR	Dichotomous	NO	0.05	NR	0.2	0.35	138	Lit	1.71	0.32
Boogaard et al, 2007	2007	NR	Continuous	NO	0.05	two-sided	0.2	6.3	180	Lit	0.4	NR
Boyer et al, 2009	2009	NCT00685230	Dichotomous	NO	0.05	two-sided	0.2	0.2	14	OE	4.75	0.16
Cattarelli et al, 2006	2006	NR	Continuous	NO	0.05	two-sided	0.1	NR	40	NR	NR	NR
Chhabra et al, 2009	2009	NR	Dichotomous	NO	0.05	NR	0.1	0.5	200	PS	0.54	0.479
Choong et al, 2010	2010	NR	Continuous	NO	0.05	NR	0.1	NR	30	OE	NR	NR
Chu et al, 2006	2006	NR	Continuous	NO	0.05	NR	0.1	0.8	40	NR	1.06	NR
Cignacco et al, 2008	2008	NR	Continuous	NO	0.05	one-sided	0.2	NR	100	NR	0.5	NR
Cogen et al, 2011	2011	NR	Continuous	NO	NR	NR	NR	NR	NR	NR	NR	NR
Cresi et al, 2008	2008	NR	Continuous	NO	0.05	NR	NR	NR	NR	NR	NR	NR
Darnell et al, 2008	2008	NCT00286052	Continuous	NO	0.05	NR	0.2	47	124	OE	-0.5	44
Doyle et al, 2007	2007	NR	Dichotomous	NO	0.05	two-sided	0.2	0.5	814	NR	1.2	0.59
Duman et al, 2010	2010	NR	Dichotomous	NO	0.05	NR	NR	NR	NR	Lit	NR	0.52
Erol et al, 2010	2010	NR	Continuous	NO	0.05	NR	0.2	NR	32	NR	NR	NR
Eslami et al, 2009	2009	NR	Continuous	NO	0.05	NR	NR	NR	NR	NR	NR	NR
Ferreira et al, 2009	2009	NR	Unclear	NO	0.05	NR	NR	NR	NR	NR	NR	NR
Fujii et al, 2010	2010	NCT00767039	Continuous	NO	0.05	NR	0.2	NR	68	Lit	NR	NR
Galante et al, 2008	2008	NR	Continuous	NO	0.05	two-sided	0.2	0.84	46	PS	2	NR
Ghai et al, 2009	2009	NR	Dichotomous	NO	0.05	NR	0.2	0.7	42	PS	0.43	0.77
Ghai et al, 2009 (2)	2009	NR	Dichotomous	NO	0.05	two-sided	0.1	0.6	112	PS	0.5	0.69
Gounaris et al, 2010	2010	NR	Continuous	YES	NR	NR	NR	NR	NR	NR	NR	NR
Gutmann et al, 2006	2006	NR	Continuous	NO	0.05	NR	0.1	NR	90	Lit	NR	NR
Hassid et al, 2007	2007	NR	Dichotomous	NO	0.05	NR	0.2	0.75	38	OE	0.33	0.39
Jacomo et al, 2011	2011	NCT00829842	Dichotomous	NO	0.05	NR	0.2	0.31	160	OE	0.5	0.298
Jeschke et al, 2007	2007	NR	Unclear	NO	0.05	NR	NR	NR	NR	NR	NR	NR
Kaabachi et al, 2007	2007	NR	Dichotomous	NO	0.05	two-sided	0.1	0.37	82	Lit	0.22	0.22
Kaabachi et al, 2007 (2)	2007	NR	Continuous	NO	0.05	two-sided	0.1	40	84	Lit	0.74	49.58
Karanovic et al, 2006	2006	NR	Dichotomous	NO	0.05	NR	0.2	NR	120	Lit	NR	NR
Khalifa et al, 2009	2009	NR	Continuous	NO	0.05	NR	NR	NR	NR	NR	NR	NR
Kneyber et al, 2008	2008	ISRCTN86554663	Continuous	NO	0.05	one-sided	0.2	84	100	Lit	0.59	54.3
Koruk et al, 2010	2010	NR	Dichotomous	NO	0.05	NR	NR	NR	18	OE	NR	NR
Kuhn et al, 2009	2009	NCT00213759	Dichotomous	NO	0.05	NR	0.1	0.4	200	Lit	0.5	0.67
Lago et al, 2008	2008	NR	Dichotomous	NO	0.05	two-sided	0.15	0.7	54	PS	0.43	NR
Lee et al, 2010	2010	NR	Dichotomous	NO	0.05	two-sided	0.2	0.85	288	Lit	0.76	0.93
Lemyre et al, 2007	2007	NR	Continuous	NO	0.05	two-sided	0.2	4.7	130	Lit	-0.5	4.7
Lucas da Silva et al, 2007	2007	NR	Continuous	NO	0.05	NR	NR	NR	NR	NR	NR	NR
Marraro et al, 2007	2007	NR	Continuous	NO	0.05	NR	NR	NR	NR	NR	NR	NR

Michel et al, 2010	2010	NCT00420693	Continuous	NO	0.05	NR	0.2	20	58	NR	0.75	NR
Murugesan et al, 2008	2008	NR	Continuous	NO	0.05	NR	NR	NR	NR	NR	NR	8.84
Nagdeve et al, 2006	2006	NR	Continuous	NO	NR	NR	NR	NR	NR	NR	NR	NR
Namachivayam et al, 2006	2006	NR	Dichotomous	NO	0.05	two-sided	0.17	0.9	40	OE	0.67	0.71
Nyman et al, 2006	2006	NR	Dichotomous	NO	0.05	NR	0.1	0.25	110	OE	0.2	0.48
Nyman et al, 2011	2011	NR	Dichotomous	NO	0.05	NR	0.1	0.8	80	Lit	0.5	0.725
Parikh et al, 2007	2007	NR	Dichotomous	NO	0.05	two-sided	0.16	0.22	120	OE	0.2	0.3
Pasha et al, 2008	2008	NR	Continuous	NO	NR	NR	NR	NR	NR	NR	NR	NR
Pedreira et al, 2009	2009	NR	Continuous	NO	0.05	NR	NR	NR	NR	NR	NR	NR
Penido et al, 2011	2011	NR	Dichotomous	NO	0.05	two-sided	0.2	NR	20	NR	NR	0.3
Pourarian et al, 2008	2008	NR	Dichotomous	NO	NR	NR	NR	NR	20	NR	NR	NR
Riethmueller et al, 2006	2006	NR	Dichotomous	NO	0.05	one-sided	0.2	0.2	120	NR	0.25	0.097
Santos et al, 2007	2007	NR	Continuous	NO	0.05	NR	NR	NR	NR	NR	NR	NR
Schroeder et al, 2010	2010	NCT00779558.	Dichotomous	NO	0.05	two-sided	0.2	0.2	160	Lit	0.25	0.16
Shi et al, 2008	2008	NR	Dichotomous	NO	0.05	NR	NR	NR	NR	NR	NR	0.42
Simons et al, 2006	2006	NR	Continuous	NO	0.05	two-sided	0.1	NR	140	NR	0.55	NR
Singh et al, 2010	2010	NR	Dichotomous	NO	0.05	NR	0.1	NR	40	Lit	NR	0.2
Sinha et al, 2010	2010	NR	Dichotomous	NO	0.05	NR	0.2	0.4	60	NR	0.25	0.533
Soltesz et al, 2007	2007	NR	Dichotomous	NO	0.05	two-sided	0.2	0.66	58	Lit	0.5	0.2
Sorce et al, 2009	2009	NR	Dichotomous	YES	0.05	NR	0.2	0.15	188	NR	NR	0.06
Uslu et al, 2010	2010	NR	Continuous	NO	0.05	NR	NR	NR	NR	NR	NR	NR
Van Woensel et al, 2011 A	2011	ISRCTN13421709	Continuous	NO	0.05	two-sided	0.2	4	128	Lit	0.5	NR
Van Woensel et al, 2011 B	2011	ISRCTN13421709	Continuous	NO	0.05	one-sided	0.2	4	102	Lit	0.5	NR
Vargas-Origel et al, 2010	2010	NR	Continuous	NO	0.05	NR	0.2	NR	NR	Lit	NR	NR
Vlasselaers et al, 2009	2009	NCT00214916	Continuous	NO	0.05	two-sided	0.2	NR	700	Lit	NR	NR
Williams et al, 2008	2008	NR	Continuous	NO	0.05	NR	0.2	NR	40	Lit	NR	114.11
Xia et al, 2011	2011	NR	Continuous	NO	0.05	NR	NR	NR	NR	NR	NR	NR
Yildizdas et al, 2008	2008	NR	Continuous	NO	0.05	NR	NR	NR	NR	NR	NR	NR
